# IgE-mediated coconut allergy in tropical Singapore

**DOI:** 10.5415/apallergy.0000000000000175

**Published:** 2025-01-08

**Authors:** Lynette Liling Tan, Si Hui Goh, May Ping Lee, Wenyin Loh, Anne Goh, Kok Wee Chong

**Affiliations:** Allergy Service, Department of Paediatric Medicine, KK Women’s and Children’s Hospital, Singapore

**Keywords:** Cocos, food hypersensitivity, Singapore

## Abstract

**Background:**

Coconut (*Cocos nucifera*) is a fruit belonging to the Arecaceae plant family. It is not a common allergen and literature on it is limited. Currently, there is a lack of data on coconut allergy in Asia, despite it being a fruit commonly used as a food ingredient in tropical Southeast Asia.

**Objective:**

In this study, we aimed to describe the demographics, clinical features, and natural history of Singaporean children with IgE-mediated coconut allergy.

**Methods:**

A retrospective review was conducted for children diagnosed with coconut allergy in a tertiary pediatric hospital in Singapore between 2017 and 2022.

**Results:**

The diagnosis of coconut allergy was made in 41 patients based on convincing history of IgE-mediated allergic reaction and a positive test (prick-to-prick test to coconut water, coconut flesh, and/or specific immunoglobulin E). The median age at first reaction was 20 months (range: 6–96 months) with most reacting on first ingestion (80.5%). Majority presented with cutaneous reactions (90.2%). Anaphylaxis occurred in 9.8%, with all involving cutaneous and respiratory systems. Most reacted to coconut milk (34.1%). Majority (82.9%) had another food allergy and a personal history of atopy (90.2%). Median duration of follow-up was 35 months (range: 3–109). Only 1 of the 41 patients reported natural tolerance at 76 months.

**Conclusions:**

Although a relatively uncommon food allergy, coconut allergy is a significant problem as anaphylaxis occurs in 1 in 10 and appears to be a persistent allergy.

## 1. Introduction

Coconut (*Cocos nucifera*) is a fruit, not a nut, belonging to the Arecaceae plant family [1]. It is not a common allergen and in many countries including Singapore, it does not need to be declared on food labels [2]. In 2023, Warren et al. reported the prevalence of coconut allergy in children in the United States to be 0.22% based on a convincing history derived using a food allergy prevalence survey. However, less than half of these children reported a physician-confirmed diagnosis [3]. Literature on coconut allergy worldwide is limited to mainly case reports and case series. The 2 largest studies published to date are both from the United States. The first study published by Kruse et al. [4] in 2021 is a 15-year retrospective study looking at 275 patients who tested positive for coconut with skin prick test (SPT) or specific immunoglobulin E (sIgE), of which 69 were allergic. The second study is the cross-sectional study published by Warren et al. [3] in 2023, which reported the clinical characteristics of 119 children with a convincing history of coconut allergy. Currently, there is a lack of data on coconut allergy in Asia, despite it being a fruit commonly used as a food ingredient in tropical Southeast Asia. Hence, this 5-year retrospective study aims to describe the demographic, clinical features, and natural history of Singaporean children with IgE-mediated coconut allergy.

## 2. Methods

The study was approved by SingHealth Centralised Institutional Review Board, Singapore (Chairperson A/Prof Agnes Ng) on July 21, 2022 (reference number: 2022/2309). Waiver of informed consent was approved by the board for this study.

Children ≤18 years old with coconut allergy diagnosed by an allergist between 2017 and 2022 were identified from our departmental electronic database at KK Women’s and Children’s Hospital, the largest tertiary pediatric hospital in Singapore. The diagnosis of coconut allergy was made based on a convincing history of an immediate allergic reaction and a positive SPT or sIgE to coconut or failed oral food challenge (OFC). Clinical records were reviewed and data on demographics, age at first reaction, type of reaction, food reacted to, concomitant food allergies, and personal and family history of atopy were collected.

SPT was performed using Stallergenes Greer Laboratories commercial extracts and a Duotip-Test II disposable skin applicator. The commercial extract was only available in our unit from December 2022. Prior to this, a prick-to-prick test (PPT) was performed on coconut water and fresh coconut flesh. Positive SPT was defined as a mean wheal diameter ≥ 3 mm after 15 minutes in comparison to the negative control. Coconut-sIgE was measured using the ImmunoCAP system FEIA (Phadia AB, Uppsala, Sweden).

Open OFC was performed at the Allergy Outpatient Specialist Clinic in our unit following the recommendations set out by the PRACTALL Consensus Report [5]. The diagnosis of anaphylaxis is in accordance with the World Allergy Organisation Anaphylaxis Guidance 2020 [6].

Data were extracted for statistical analysis using IBM SPSS Statistics ver.21 (IBM Co. Armonk, NY, USA). Demographic data were described using proportion, parametric continuous data were described as mean and standard deviation, and nonparametric continuous data were described as median and interquartile range. Contingency tables were analyzed using Fisher exact test. SPT levels between groups were compared using the Mann-Whitney test. A *P* value <0.05 was considered statistically significant.

## 3. Results

A total of 41 patients were diagnosed with IgE-mediated coconut allergy over the 5-year review period. Their demographics are shown in Table 1. Personal history of atopy was observed in 90.2% of the cohort and 82.9% had at least one other food allergy.

**Table 1.** Demographics of subjects in our study (N = 41)

	Total (N = 41)	Mild-moderate coconut allergy (n = 37)	Coconut anaphylaxis (n = 4)
Age at presentation, median (range), y	3.5 (0.9–16.0)	3.5 (1.0–16.0)	2.7 (0.9–4.5)
Gender, male	30 (73.2%)	26 (70.3%)	4 (100.0%)
Ethnicity			
Chinese	19 (46.3%)	17 (45.9%)	2 (50.0%)
Malay	11 (26.8%)	10 (27.0%)	1 (25.0%)
Indian	8 (19.5%)	8 (21.6%)	0 (0.0%)
Others	3 (7.3%)	2 (5.4%)	1 (25.0%)
Personal history of atopy	37 (90.2%)	33 (89.2%)	4 (100%)
Allergic dermatitis	36 (87.8%)	33 (89.2%)	3 (75.0%)
Atopic rhinitis	22 (53.7%)	21 (56.8%)	1 (25.0%)
Asthma	9 (22.0%)	9 (24.3%)	0 (0.0%)
Other food allergies/sensitizations	34 (82.9%)	31 (83.8%)	3 (75.0%)
Egg	22 (53.7%)	20 (54.1%)	2 (50.0%)
Peanut	21 (51.2%)	19 (51.4%)	2 (50.0%)
Tree nuts	20 (48.8%)	18 (48.6%)	2 (50.0%)
Almond	7	5	2
Hazelnut	6	4	2
Cashew	17	15	2
Pistachio	14	13	1
Walnut	5	5	0
Pecan	3	3	0
Brazil nut	8	6	2
Cow’s milk	12 (29.3%)	10 (27.0%)	2 (50.0%)
Shellfish	7 (17.1%)	6 (16.2%)	1 (25.0%)
Family history of atopy	23 (56.1%)	20 (54.1%)	3 (75.0%)

Table 2 shows the clinical features of allergic reactions to coconut ingestion. The median age at first reaction was 20 months (range: 6–96) with the majority reacting on first exposure (80.5%). The majority presented with cutaneous manifestations (90.2%). Four children had anaphylaxis presenting with cutaneous symptoms and respiratory compromise. None had cardiovascular involvement. The first reacted to coconut milk, the second to coconut yogurt, the third to fresh coconut, and it was not clear the form of coconut for the last child. Severity of reaction was not significantly associated with SPT wheal size or other variables such as age at first reaction, concurrent food allergies, personal history of atopy, and family history of atopy.

**Table 2.** Clinical features of allergic reaction to coconut (N = 41)

Age at first reaction, median (range), mo	20 (6–96)
Number who reacted on first ingestion	33 (80.5%)
Food that children first reacted to	
Coconut milk	15 (36.6%)
Desiccated coconut	6 (14.6%)
Coconut yogurt/ice cream	6 (14.6%)
Fresh coconut	4 (9.8%)
Coconut water	4 (9.8%)
Coconut oil	2 (4.9%)
Unknown	4 (9.8%)
Clinical manifestations	
Cutaneous	37 (90.2%)
Gastrointestinal	6 (14.6%)
Respiratory	4 (9.8%)
Cardiovascular	0 (0.0%)
Anaphylaxis	4 (9.8%)
Skin prick test, median (25th–75th percentile), mm	
Coconut water	9.0 (5.5–11.0)
Coconut flesh	9.5 (7.0–10.5)
sIgE, median (25th–75th percentile), kU/L	25.7 (11.6–39.8)

sIgE, specific immunoglobulin E.

Most patients (37/41) had positive PPT to coconut flesh and/or coconut water. One patient had a negative PPT to both coconut water and coconut flesh but had a positive PPT to coconut milk. Six had positive sIgE. Three had positive sIgE only and 3 had both a positive PPT and sIgE. OFC was only performed for 1 patient. He passed a coconut water challenge but reacted during a coconut flesh challenge.

Patients most commonly first reacted to coconut milk (Table 2). Data on tolerance to coconut water were known in 13 patients, with 8 reporting tolerance. Data on topical use of coconut-containing products were known in 9 patients, with 7 having used such products prior to diagnosis of coconut allergy.

Six patients were lost to follow-up after the first visit. The median follow-up for the remaining 35 patients was 35 months (range: 3–109). The majority (27/35) of these patients had repeat skin testing on follow-up visits. Only 1 of the 41 patients reported natural tolerance at 76 months via successful home introduction. Another patient had a negative SPT and sIgE at 52 months and was advised for the home introduction of coconut but had not done so at the last visit.

## 4. Discussion

Coconut is not a common allergen but is of importance especially in Asia as it is a frequently used food ingredient. Kruse et al. [4] reported that Asian children had a 2-fold increased rate of coconut allergy versus sensitization. Till date, this is the largest report of coconut allergy in Asia. Children in our cohort presented young with a median age of first reaction of 20 months. Wongnate et al. [7] recently reported 11 Thai children diagnosed clinically with coconut allergy, who also had an early onset of allergy (median age of 12 months). This early onset of allergy in Asia contrasts with the West with a mean age of first reaction of 4 years old in the Australian cohort and 5 years old in the American cohort [4, 8]. This may be due to the earlier exposure in Asian children due to the ubiquity of coconut in our diet. Anaphylaxis occurred in 9.8% of our study cohort, compared to 27.2% in the Thai cohort, 25.7% in the Australian cohort, and 24.5% in the American cohort [4, 7, 8]. Although our anaphylaxis rate was not as high as the other cohorts, it is still higher compared to anaphylaxis due to other food allergies in our Singaporean children (cow’s milk: 5.1%, peanut: 7.1%, cashew: 3.8%, and fish: 6.5%) [9–12].

Coconut (*Cocos nucifera*) is a fruit native to the coastal areas of Southeast Asia and Melanesia and is currently found throughout the tropics and subtropics. It consists, from the outside in, of a hard skin (exocarp), fibrous husk (mesocarp), hard shell (endocarp), white kernel (endosperm), and a large cavity filled with liquid (water). The water can be drunk, and the kernel can be eaten fresh or shredded and dried. Coconut milk and coconut oil are also extracted from the kernel. Desiccated coconut, coconut milk, and coconut oil are used in a variety of savory and sweet dishes [13]. Popular local dishes that use coconut include “nasi lemak” (a fragrant rice dish cooked in coconut milk), “laksa” (coconut curry noodle dish), curry, “cendol” (iced sweet dessert containing coconut milk), and “kueh” (bite-sized Southeast Asian snacks) [14]. Traditionally, coconut water has long been a popular cooling drink in our hot climate and in recent years, coconut milkshake has also become popular. Besides food, coconut is also ubiquitous in skin care products. A 2004 study found that coconut was the most common food found in commercially available pediatric skin care products and a more recent study in 2017 found that the most popular moisturizer based on the number of reviews at 3 major online retailers was a brand of coconut oil [15, 16].

Coconut allergens include the seed storage proteins Coc n 1 (7S globulin), Coc n 2 (7S globulin), and Coc n 4 (11S globulin), with the former being the only coconut allergen included in the official allergen nomenclature subcommittee of the International Union of Immunological Societies [1, 17]. In the literature, most cases reacted to foods containing fresh coconut, desiccated coconut, coconut milk, or coconut cream, with coconut milk most reported [1, 8, 17–25]. Only 2 cases reacted to coconut water [8] and 3 cases reacted to coconut oil [1, 17, 26]. This finding is similar to our study with the majority reacting to coconut milk or flesh, and <15% reacting to coconut water and coconut oil. This is most likely related to the amount of protein found in different forms of coconut. Both coconut water (0.72 g vs 2.29 g per 100 g, respectively [8]) and coconut oil (16–26 mg/mL vs 41 mg/mL, respectively [17]) contain less protein compared with coconut milk. The refining process also affects the protein content of coconut oil, with unrefined coconut oil previously been shown to have a much higher protein content compared with refined coconut oil (251 mg/mL vs 7.9 mg/mL, respectively) [27]. Cooking coconut milk also affects the protein content, with boiled coconut milk containing less protein compared with fresh coconut milk (24 mg/mL vs 41 mg/mL, respectively) [17].

Patients with nut allergy may sometimes also avoid coconut for concerns of cross-reactivity. However, literature on cosensitization between coconut and tree nuts remains conflicting. Previous case reports have described cross-reactivity between coconut and tree nuts (hazelnut, walnut) being likely due to seed storage proteins [21, 22, 28]. Polk et al. [29] also found that children with almond and macadamia sensitization were more likely to be sensitized to coconut. This is supported by the study by Kruse et al. [4] showing that cosensitization with tree nuts was common, with macadamia having the strongest correlation with coconut. It is important to remember that cosensitization does not equate to clinical reactivity. On the other hand, the study by Stutius et al. [30] with 40 children found that patients with peanut or tree nuts sensitization or allergy were not more likely to be sensitized or allergic to coconut. This is in keeping with the study by Pathmanandavel et al. [8] of 35 children showing that the incidence of coconut allergy is not higher in children with tree nut allergy compared with other food allergies. In our cohort, about half had a concomitant peanut or tree nut sensitization or allergy with peanut, cashew, and pistachio most seen. We believe this finding suggests an overall predisposition to food allergies and are unable to conclude whether children with coconut allergy are more likely to be allergic to peanut and tree nuts.

Not much is known about the natural history of coconut allergy. Only 1 of the 69 coconut allergic patients acquired natural tolerance in the Kruse et al. study [4] and in the Warren et al. study, 31.8% of children with a history of convincing coconut allergy self-reported outgrowing their allergy. Overall, it appears to be low and, in our study, only one patient was found to acquire natural tolerance at 76 months. Kruse et al. [4] found that an SPT of 9 mm or sIgE of 58 kU/L had a 95% probability of reaction. About half of our patients had tests that were below these thresholds at follow-up, but OFCs were not performed and hence we may be underestimating our outgrowing rate. The wide follow-up range from 3 to 109 months may also affect this as some patients may not have been seen long enough to know if they have outgrown their allergy.

With the rising popularity of coconut-containing skin care products, there have been concerns about the risk of cutaneous sensitization and subsequent development of allergy as described in the dual-allergen exposure hypothesis, especially in children with atopic dermatitis [31]. This data was known in 57 of the 275 children in the study by Kruse et al. [4], with about two-thirds of these patients with coconut sensitization or allergy reporting a history of topical use of coconut. In our study, 7 of 9 patients with known history of topical coconut use reported using such products prior to diagnosis of coconut allergy. In Singapore, a consensus statement published in 2019 on the primary prevention of allergy in at-risk infants recommended that moisturizers should not contain food products [32]. Although more robust studies are required to prove these casual relationships, we would still advise against the use of coconut-containing topical products in young infants, before confirming oral tolerance.

Limitations of our study include its retrospective design and lack of OFC for the diagnosis of coconut allergy and assessment of tolerance. Clinically, the diagnosis of food allergy can be made when there is a convincing history of an immediate allergic reaction and a positive SPT or sIgE. Hence, an OFC is usually required only if the history or test results are equivocal. More OFCs should have been performed for the assessment of tolerance at follow-up, but some families were content with continued avoidance. Our duration of follow-up may not have been sufficiently long to understand the true outgrowing rates of coconut allergy. There was also a lack of standardized skin testing prior to 2022. However, in a few patients who had testing done using both the commercial extract and PPT in the same setting, the results were similar and we do not think it would significantly affect our management. We have now standardized skin testing in our center using the commercial coconut extract as we feel it is equivalent to doing a PPT and parents would not need to bring coconut for testing. There was also incomplete data on topical use of coconut-containing products.

## 5. Conclusions

Although a relatively uncommon food allergy, coconut allergy is a significant problem as anaphylaxis occurs in 1 in 10 and appears to have low rates of outgrowing. Concomitant food allergy is prevalent among children with coconut allergy and further work needs to be done to clarify the cosensitization patterns between coconut and tree nuts. More studies are also required to confirm the causal relationship between topical coconut application and the development of coconut allergy.

## Acknowledgements

We thank our allergy specialist nurses, Ms Lim Hwee Hoon, Ms Sindhu Raveendran, and Ms Kaira How Kai Li, and our SPT laboratory technicians for their hard work.

## Conflicts of interest

The authors have no financial conflicts of interest.

## Author contributions

Lynette Liling Tan and Kok Wee Chong: conceptualization and design. Lynette Liling Tan: writing—original draft. Si Hui Goh, May Ping Lee, Wenyin Loh, Anne Goh, and Kok Wee Chong: writing—review and editing.
